# Discrepancies on the Role of Oxygen Gradient and Culture Condition on Mesenchymal Stem Cell Fate

**DOI:** 10.1002/adhm.202002058

**Published:** 2021-02-02

**Authors:** Jay R. K. Samal, Vignesh K. Rangasami, Sumanta Samanta, Oommen P. Varghese, Oommen P. Oommen

**Affiliations:** ^1^ Department of Instructive Biomaterial Engineering MERLN Institute for Technology‐Inspired Regenerative Medicine Maastricht University Maastricht 6229 ER The Netherlands; ^2^ Bioengineering and Nanomedicine Group Faculty of Medicine and Health Technologies Tampere University Tampere 33720 Finland; ^3^ Translational Chemical Biology Laboratory Department of Chemistry, Polymer Chemistry Ångström Laboratory Uppsala University Uppsala 751 21 Sweden

**Keywords:** differentiation, hypoxia, mesenchymal stem cells, oxygen gradients, proliferation

## Abstract

Over the past few years, mesenchymal stem (or stromal) cells (MSCs) have garnered enormous interest due to their therapeutic value especially for their multilineage differentiation potential leading to regenerative medicine applications. MSCs undergo physiological changes upon in vitro expansion resulting in expression of different receptors, thereby inducing high variabilities in therapeutic efficacy. Therefore, understanding the biochemical cues that influence the native local signals on differentiation or proliferation of these cells is very important. There have been several reports that in vitro culture of MSCs in low oxygen gradient (or hypoxic conditions) upregulates the stemness markers and promotes cell proliferation in an undifferentiated state, as hypoxia mimics the conditions the progenitor cells experience within the tissue. However, different studies report different oxygen gradients and culture conditions causing ambiguity in their interpretation of the results. In this progress report, it is aimed to summarize recent studies in the field with specific focus on conflicting results reported during the application of hypoxic conditions for improving the proliferation or differentiation of MSCs. Further, it is tried to decipher the factors that can affect characteristics of MSC under hypoxia and suggest a few techniques that could be combined with hypoxic cell culture to better recapitulate the MSC tissue niche.

## Graphical abstract

This progress report surveys the literature and discusses the conflicting results on the effect of oxygen exposure during cell culture that affect mesenchymal stem cell function and few promising strategies are suggested that when combined with hypoxic culture would facilitate consistent results.

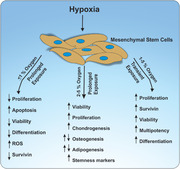

## 1 Introduction

Albeit there has been a significant increase in the lifespan of humans over the past few decades, there has also been a corresponding increase in several ailments, which can severely impact the standard of living. These ailments may be overcome by tissue engineering strategies, which involve regeneration of tissues using stem cells, biomaterials and growth factors.^[^ ^1^ ^]^

Stem cells are generally used for tissue engineering applications and have the defining characteristic to self‐renew or differentiate into specialized cells depending on the conditions in their environment. Based on their source, stem cells can be classified as adult stem cells, which are found throughout the body and are generally multipotent in nature; embryonic stem cells, which are obtained from the inner cell mass of the developing blastocyst and are pluripotent in nature; and induced pluripotent stem cells that are engineered by genetic reprogramming of a fully differentiated cell such as fibroblast into a pluripotent embryonic stem cell‐like state.^[^ ^2^ ^]^

Over the past few years, mesenchymal stem/stromal cells (MSCs) have garnered considerable interest due to their therapeutic value and potential application in tissue engineering strategies.^[^ ^3^ ^]^ MSCs are adult multipotent stem cells capable of differentiating into various tissues such as bone, muscle, cartilage and fat, among others.^[^ ^3^, ^4^ ^]^ However, the application of MSCs is still limited due to low rates of proliferation and differentiation in vitro.^[^ ^5^ ^]^ The development of functional tissue constructs using MSCs is further restricted by the limited understanding of the complex in vitro conditions required for maintaining the desired cellular characteristics’ and preventing senescence.^[^ ^6^ ^]^ Moreover, these cells are exposed to a harsh cellular microenvironment during transplantation, which can lead to cellular damage.^[^ ^7^ ^]^ Many approaches have been proposed to mimic the native cellular microenvironment experienced by MSCs in a bid to improve their cell number and differentiation potential in vitro. These approaches include the addition of various growth factors and culturing cells on suitable substrates to provide appropriate physiochemical cues.

One of these approaches has been the application of hypoxia during MSC culture. (We used the terms physioxia for oxygen gradient in normal tissue present under in‐vivo conditions; hypoxia for low oxygen in‐vitro cell culture conditions and normoxia for ambient in‐vitro cell culture conditions). As low oxygen (O_2_) concentrations (physioxic conditions or hypoxic conditions) have been observed in the tissue niches where MSCs reside in vivo, it has been suggested that hypoxia could be applied to MSCs in vitro to recapitulate the influence of native local signals on differentiation or proliferation of these cells. Hypoxia represents a physiological stimulus that triggers various signaling pathways within a cell and can lead to either cell death or cell adaptation.^[^ ^7^ ^]^

Can hypoxic cultures affect MSC cell fate? What are the factors that can affect cells under hypoxia? Previous reviews have excellently described the molecular mechanisms and signaling pathways involved in the role of hypoxia on the regulation of stem cell biology in general,^[^ ^8^ ^]^ as well as the effect of hypoxia on the regulation of MSC biology and formation of mesenchymal tissues, in particular.^[^ ^9^ ^]^ These reviews provide an in‐depth understanding of the mechanistic effect of hypoxia on stem cells and on the underlying cellular responses. Our aim in this review is to present the conflicting results reported on MSCs differentiation as a response to hypoxic cell culture conditions. We make an earnest attempt to rationalize the variable results to variable factors and culture conditions that have different consequences on these cells and skew the effect of hypoxia on these cells.

## 2 MSCs and Hypoxia

### 2.1 MSCs Experience Low Oxygen Concentration In Vivo

**Figure 1:**
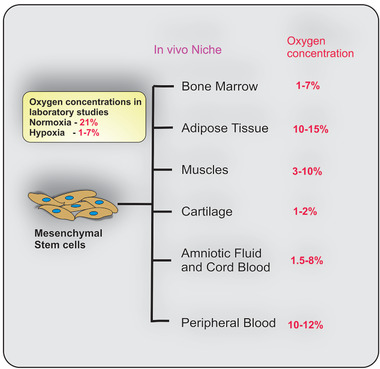
Mesenchymal stem cells (MSCs) experience low oxygen (O_2_) concentrations in vivo. Depending on the in vivo niche, MSCs may experience low O_2_ concentrations ranging from 1–15%.

In vivo, MSCs are found in the bone marrow, adipose tissue, muscles, amniotic fluid, umbilical cord blood and peripheral blood.^[^ ^3^, ^10^ ^]^ Depending on the in vivo niche, MSCs may experience low O_2_ concentrations, even lower than 1% (**Figure** **1**).^[^ ^5^, ^11^ ^]^ For example, the O_2_ concentration experienced by MSCs in the bone marrow varies from 1–7%,^[^ ^12^ ^]^ 10–15% in adipose tissue,^[^ ^13^ ^]^ 3–10% in muscles,^[^ ^14^ ^]^ 1–2% in cartilage,^[^ ^15^ ^]^ 1.5–8% in amniotic fluid and umbilical cord blood,^[^ ^16^ ^]^ and 10–12% in peripheral blood.^[^ ^11^ ^]^ This physiological O_2_ concentration (physioxia) is markedly lower than the 21% O_2_ found in normoxic conditions generally used for MSC culture in laboratories.^[^ ^5^ ^]^ It has been suggested that this difference in O_2_ concentrations may lead to higher free radical generation in normoxia, which could impair the functioning of MSCs.^[^ ^17^ ^]^

Various studies involving the application of hypoxia to MSCs show highly variable results, which could be due to the variation in the O_2_ concentration considered to be hypoxia between the different studies, with values ranging from 1–7%.^[^ ^18^ ^]^ The variable results are further compounded by differences in culture conditions, selection markers, supplements and growth factors between the studies.^[^ ^9a^ ^]^ These studies generally involve either a) expansion in normoxia and exposure to hypoxia during differentiation or b) expansion in hypoxia and differentiation in normoxia.

### 2.2 Hypoxic Conditions can Enhance MSC Proliferation and Multipotency

**Figure 2:**
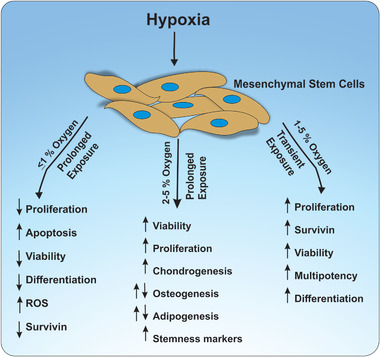
Effect of hypoxia on MSC fate. Depending on the oxygen concentration and duration of exposure to hypoxia, variable effects have been reported. While prolonged exposure of more than 24 h to acute hypoxia (≤1% O_2_) is reported to reduce MSC proliferation and increase apoptosis, prolonged exposure to 2–5% O_2_ shows increased chondrogenesis and proliferation, with both promoting and ameliorating effects reported on osteogenesis and adipogenesis. Transient exposure of MSCs to 1–5% O_2_ can lead to the upregulation of multipotency and proliferation. Increased osteogenic and adipogenic differentiation potential has been reported for subsequent differentiation of hypoxia pre‐treated MSCs under normoxia.

Enhanced proliferative and colony‐forming potential of both human and mouse MSCs has been reported for O_2_ concentrations ranging from 1–5%, when compared to cells cultured in normoxia (**Table** **1**, **Figure** **2**).^[^ ^5^, ^12^, ^19^ ^]^ It has been suggested that the increase in proliferation could be due to down regulation of p16, leading to escape from senescence.^[^ ^19a^ ^]^

When exposed to hypoxia, human mesenchymal stem cells (hMSCs) initially exhibit enhanced cell death, within the first 1–2 h, along with impairment of various cellular functions.^[^ ^7^ ^]^ In this state, hMSCs may undergo autophagy, which could be an initial response to hypoxia, as a survival mechanism.^[^ ^20^ ^]^ However, increased proliferation rates have been observed with increased duration of hypoxic exposure (at 2–5% O_2_ concentration).^[^ ^5^, ^12^, ^21^ ^]^ Along with downregulation of p16, hypoxia also leads to the stabilization of hypoxia‐inducible factor (HIF), which can result in the induction of a multitude of signaling pathways within the cell, notably an increase in the expression levels of the antiapoptotic protein survivin, thus contributing to improved proliferation.^[^ ^22^ ^]^

In addition to enhanced proliferation, human and murine MSCs cultured under hypoxic conditions (at 2–5% O_2_ concentration), also display upregulation of multipotency, observed by upregulation of stemness related genes such as Oct‐4, Sox2, and Nanog, and early mesodermal genes.^[^ ^12^, ^19^, ^23^ ^]^ This has prompted the suggestion that MSCs could retain characteristics of “true stem cells” under hypoxia as the undifferentiated state is maintained, along with upregulation of genes related to mesodermal and non‐mesodermal lineages.^[^ ^7^, ^23^ ^]^

hMSCs expanded at 2% O_2_ have been shown to yield higher cell numbers but no difference in adipogenic or osteogenic differentiation could be observed between hypoxic and normoxic cells during differentiation under normoxia, while cells expanded at 3% O_2_ showed higher differentiation under normoxia.^[^ ^12^, ^19^ ^]^ Furthermore, long term culture of hMSCs of upto 4 passages, at 5% O_2_ has been shown to promote maintenance of the undifferentiated state of the cells, along with increased proliferation in basal medium. However, post‐expansion in hypoxia, these cells showed higher potential for osteogenic differentiation in normoxia, compared to cells expanded under normoxia, suggesting augmentation of multipotency.^[^ ^23c^ ^]^

**Table 1:** Summarizes studies involving analysis of the proliferation potential of mesenchymal stem cells under hypoxia

Reference	Material	Conditions and device used	Cell Source	Medium/Growth Factors Used	Reported Result
Wu et al.^[^ ^20^ ^]^	6‐well or 24‐well plates	Pre‐conditioning with hypoxia (5% O_2_) for 6 h in a hypoxia chamber	Mouse bone marrow‐derived mesenchymal stem cells (mbMSC)	Basal medium (DMEM supplemented with 15% FBS, 2 × 10^−3^ m L‐glutamine and antibiotics) for expansion	Hypoxia induces autophagy and LC‐3 expression in mbMSCs.
Burian et al.^[^ ^24^ ^]^	2D cell culture flask	Hypoxic culture (2% O_2_) in humidified incubator (MCO‐5M, Sanyo) for 21 days;	Porcine bone marrow‐derived MSC (pbMSC) and porcine adipose‐derived MSC (paMSC) Cell density 5 × 10^3^ cells mL^−1^	Basal medium for expansion (*α*‐MEM supplemented with 10% FBS and antibiotics)	More homogenous proliferation in hypoxic cultured pbMSC and paMSC.
Boyette et al.^[^ ^5^ ^]^	6‐well or 24‐well plates	Hypoxic culture (5% O_2_) for 21 days in closed incubators	Human bone marrow‐derived mesenchymal stem cells (hbMSC) Cell density 1 × 10^4^ cells cm^−2^	Basal medium (High glucose DMEM supplemented with 10% FBS and antibiotics)	Hypoxia increased proliferation of hbMSCs in basal medium.
Lennon et al.^[^ ^17^ ^]^	Tissue culture plates	Hypoxic culture (5% O_2_) for 21 days in closed incubator chambers	Rabbit bone marrow‐derived mesenchymal stem cells (rbMSC) Cell density 5 × 10^7^ cells 100 mm^−1^	Basal medium (Low‐glucose DMEM supplemented with 10% FBS and antibiotics)	RbMSCs proliferated more rapidly in hypoxic conditions in vitro and upon transplantation produced more bone in vivo.
Basciano et al.^[^ ^23c^ ^]^	Tissue culture plates	Hypoxic culture (5% O_2_) for 4 passages in incubator (Sanyo).	hbMSC Cell density 50 × 10^3^ cells cm^−2^ for cell expansion	Basal medium (*α*‐MEM supplemented with 10% FBS, 2 × 10^−3^ m glutamine and antibiotics)	Hypoxia promoted maintenance of the undifferentiated state of hbMSCs, alongwith increased proliferation in basal medium.
dos Santos et al.^[^ ^19b^ ^]^	12‐well plates	Hypoxic culture (2% O_2_) in C‐Chamber connected to Proox Model 21 controller (BioSpherix)	hbMSC Cell density 1000 cells cm^−2^ for cell expansion	Basal medium (DMEM supplemented with 10% FBS and antibiotics)	Hypoxia promoted maintenance of differentiative potential of hbMSCs and yielded higher cell numbers and population doubling.
Fehrer et al.^[^ ^12^ ^]^	6‐well plates	Hypoxic culture (3% O_2_) in Thermo Electron Corporation 3110 incubators	hbMSC Cell density 0.2–0.5 × 10^6^ cells cm^−2^ for cell expansion	Basal medium (MEM supplemented with 20% FCS and antibiotics)	Increased proliferative lifespan, with higher number of passages of hbMSCs exposed to hypoxia. Hypoxic hbMSCs showed enhanced proliferation as compared to cells cultured in normoxia.
Wang et al.^[^ ^25^ ^]^	Alginate beads	Hypoxic culture (5% O_2_) in a low oxygen incubator (NAPCO) for 14 days.	Human adipose derived MSC (haMSC) Cell density 4 × 10^6^ cells mL^−1^	Basal medium (High glucose DMEM supplemented with 110 mg L^−1^ sodium pyruvate, 10% FBS, and antibiotics)	Hypoxia significantly reduced the proliferation of haMSCs, while increasing chondrogenic differentiation potential.
Holzwarth et al.^[^ ^26^ ^]^	96‐well plates	Hypoxic culture (5%, 3%, or 1% O_2_) for 14 days in Heracell gas addition incubators (Heraeus Instruments GmbH)	hbMSC Cell density 6250 cells cm^−2^ for cell expansion	Basal medium (Low glucose DMEM supplemented with 5% human fresh frozen plasma, 10^7^ mL^−1^ platelets, 80 IU mL^−1^ heparin sulphate, 1 × 10^−3^ m glutamine and antibiotics)	hbMSCs showed reduced proliferation and under 1% O_2_. Cells cultured at 3% O_2_ showed higher proliferation. Decline in proliferation rate and metabolic activity with reducing O_2_ concentration observed.
Ren et al.^[^ ^18^ ^]^	T25 culture flasks	Hypoxic culture (8% O_2_) for 7–8 days in modular airtight humidified chamber	mbMSC Cell density 1 × 10^5^ cells cm^−2^	Basal medium (IMDM with 10% FBS and antibiotics)	Mice bMSCs showed increased cell proliferation in basal medium under hypoxia as compared to cells cultured under normoxia.
Potier et al.^[^ ^27^ ^]^	Pellet culture for chondrogenesis	Hypoxic culture (1% O_2_) for 48 h and 120 h in sealed jar (Oxoid Ltd) containing O_2_ chelator (AnaeroGen)	hbMSCs Cell density 5000 cells cm^−2^	Basal medium (*α*‐MEM supplemented with 10% FBS and antibiotics)	Temporary exposure (48 h) to hypoxia showed no difference in hbMSC survival. Exposure to hypoxia for 120 h led to increased cell death rates.
Grayson et al.^[^ ^23d^ ^]^	Synthetic poly(ethylene terephthalate) (PET) fibrous matrices with 100 to 200 µm pore size	Hypoxic culture (2% O_2_) for 30 days in sealed chamber	hbMSCs Cell density 3 × 10^6^ cells per PET disk	Basal medium (*α*‐MEM supplemented with 10% FBS and antibiotics	hbMSCs showed increased proliferation and maintenance of undifferentiated state in basal medium under hypoxia.
Krinner et al.^[^ ^28^ ^]^	96‐well plates for clonal expansion assay	Hypoxic culture (5% O_2_) for 14 days in tri‐gas incubator (Thermo Fisher Scientific)	Sheep bone marrow derived MSC (sbMSC) Cell density 1 cell/96‐well for clonal expansion	Basal medium (High‐glucose DMEM supplemented with 10% FCS and antibiotics)	Cells cultured under hypoxia showed increased proliferation as compared to cells cultured under normoxic condition.
Hu et al.^[^ ^29^ ^]^	96‐well plates for cell proliferation	Hypoxic culture (5% O_2_ and 10% O_2_) for 14 days.	mbMSC Cell density 1 × 10^4^ cells/6‐well for proliferation	Basal medium (DMEM with 1500 mg L^−1^ D‐glucose, 20% FBS, 1% glutamine and antibiotics)	Mice bMSCs showed enhanced proliferation at 5% O_2_ compared to 10% O_2_ and normoxia.
Henrionnet et al.^[^ ^30^ ^]^	T75 tissue culture flask for expansion,	Hypoxic culture (5% O_2_) for 28 days	hbMSC Cell density 50 000 cells cm^−2^ for expansion	Basal medium (Low glucose DMEM supplemented with 10% FBS 1 ng mL^−1^ bFGF and antibiotics for expansion)	No significant change in proliferation when cells are exposed to hypoxia as compared to normoxia.

### 2.3 The Clonogenic Potential of MSCs can be Enhanced under Hypoxia

The clonogenicity of MSCs, which represents the ability of MSCs to clone themselves and subsequently form a colony of “cloned cells” can significantly influence MSC fate by modulating the proliferation and differentiation potential.^[^ ^31^ ^]^ Exposure to hypoxic conditions has shown to increase the clonogenic potential of both human and porcine bone derived MSC, consequently decreasing the differentiation potential (**Table** **2**).^[^ ^5^, ^32^ ^]^ Multiple studies have shown upregulation of vascular endothelial growth factor (VEGF) expression in MSCs upon exposure to hypoxic conditions.^[^ ^5^, ^32^, ^33^ ^]^ Moreover, increased half‐life and enhanced secretion of VEGF mRNA has been observed under hypoxic conditions.^[^ ^34^ ^]^ VEGF expression has been linked to hypoxia‐inducible factor 1, which is upregulated under hypoxia and could explain upregulated VEGF expression under hypoxia.^[^ ^35^ ^]^ It has been suggested that the increased VEGF expression directly enhances MSC colony formation.^[^ ^5^, ^32^ ^]^ Furthermore, increased cell proliferation, enhanced secretion of growth factors including VEGF, and increased matrix turnover could be factors influencing increased clonogenicity.^[^ ^5^, ^32^ ^]^ In addition, increased metabolic activity and proliferation rates were also observed, which could also contribute to enhanced clonogenicity.^[^ ^32^ ^]^

**Table 2:** Summarizes the studies that apply hypoxia for studying changes in clonogenic potential of MSCs. These studies have reported that the clonogenic potential of MSCs is enhanced under hypoxic culture conditions

Reference	Material	Conditions and device used	Cell Source	Medium/Growth Factors Used	Reported Result
Boyette et al.^[^ ^5^ ^]^	6‐well or 24‐well plates	Hypoxic culture (5% O_2_) for 21 days in closed incubators;	Human bone marrow derived MSC (hbMSC) Cell density 1 × 10^4^ cells cm^−2^	Basal medium (High glucose DMEM supplemented with 10% FBS and antibiotics) for expansion	Increase in clonogenicity due to increased cell proliferation, increased secretion of VEGF, and increased matrix turnover
Antebi et al.^[^ ^32^ ^]^	Tissue culture flasks	Hypoxic culture (1% O_2_) for long‐term (10 days) and short‐term (48 h). Or Short‐term (48 h) 2% and 5% O_2_ in hypoxia station (HypOxystation H35, HypOxygen)	hbMSC and procine bMSC Cell density 3 × 10^5^ cells cm^−2^	Basal medium (*α*‐MEM supplemented with 15% FBS. 2 × 10^−3^ m L‐glutamine, and antibiotics)	Slower proliferation and lower yields of MSCs under long term exposure to hypoxia. Short term hypoxic culture led to significantly faster proliferation. Increased clonogenic potential due to increased expression of VEGF and decreased expression of apoptotic genes BCL‐2 and CASP3 in both short and long term hypoxic exposures.
Li et al.^[^ ^36^ ^]^	Tissue culture flasks	Hypoxic culture (2.5% O_2_) in a hypoxia gas chamber for 5 days	Mouse bone marrow derived MSCs (mbMSC)	Basal medium (DMEM/F12 supplemented with 10% FBS and antibiotics)	mbMSCs exposed to hypoxia had higher cell viability and proliferation potential Hypoxic mbMSCs showed increased clonogenic potential alongwith increased cell proliferation
Krinner et al.^[^ ^28^ ^]^	96‐well plates for clonal expansion assay	Hypoxic culture (5% O_2_) for 14 days in tri‐gas incubator (Thermo Fisher Scientific)	Sheep bone marrow derived MSC (sbMSC) Cell density 1 cell/96‐well for clonal expansion	Basal medium (High‐glucose DMEM supplemented with 10% FCS and antibiotics)	Alongwith proliferation, hypoxia increased clonogenicity and colony forming ability of ovine MSCs.
Hu et al.^[^ ^29^ ^]^	6‐well plates for clonal expansion assay	Hypoxic culture (5% O_2_ and 10% O_2_) for 14 days.	mbMSC Cell density 1 × 10^4^ cells/96‐well for clonal expansion	Basal medium (DMEM with 1500 mg L^−1^ D‐glucose, 20% FBS, 1% glutamine and antibiotics)	Mice bMSCs showed enhanced clonogenicity and colony forming ability at 5% O_2_ compared to 10% O_2_ and normoxia.
Adesida et al.^[^ ^37^ ^]^	T150 tissue culture flask for clonal expansion	Hypoxic culture (3% O_2_) for 14 days	hbMSC Cell density 100 000 cells cm^−2^ for expansion	Basal medium (*α*‐MEM supplemented with 10% FBS, 4‐(2‐hydroxyethyl)‐1‐piperazineethanesulfonic acid (HEPES), sodium pyruvate, 5 ng mL^−1^ basic fibroblast growth factor (bFGF) and antibiotics)	Significantly higher number of hbMSC colonies under hypoxia as compared to normoxia.

### 2.4 Hypoxic Conditions can Modulate MSC Secretome

In addition to their differentiation potential, MSCs have also been implicated in influencing native cells at the site of damaged tissue toward wound healing via paracrine signaling through the secretome.^[^ ^38^ ^]^ Post injection into the body, MSCs migrate toward the site of damaged tissue and can inhibit secretion of pro‐inflammatory cytokines, thereby improving cell survival around the damaged tissue.^[^ ^39^ ^]^

Pre‐conditioning of MSCs with hypoxic conditions ranging from 24–72 h showed significantly stronger immunomodulatory properties of the collected secretome when compared to normoxic culture conditions (**Table** **3**).^[^ ^38^ ^]^ Further, bone marrow derived mesenchymal stem cells (bMSCs) have been shown to induce hepatocyte growth factor (HGF) and VEGF expression in medium, with enhanced secretion observed with hypoxia pre‐conditioned MSCs, with similar results observed with Wharton's jelly MSC (WJMSC) and adipose derived‐mesenchymal stem cells (ADMSCs).^[^ ^33^, ^40^ ^]^ WJMSCs are neonatal in nature and can be collected at the time of delivery.^[^ ^41^ ^]^ ADMSCs are derived from the stromal vascular fraction of adipose tissue.^[^ ^42^ ^]^ The increased VEGF secretion could enable better maintenance of stemness of hematopoietic stem cells upon co‐culture with WJMSC under hypoxia.^[^ ^40b^ ^]^ Secretome obtained from hypoxia preconditioned ADMSCs has further been shown to promote healing of the gastric mucosa in rats, as compared to normoxic culture, by enhancing angiogenesis and reepithelialisation.^[^ ^33^ ^]^

Apart from introducing the secretome from hypoxia conditioned MSCs to other cells, co‐culture of hypoxia conditioned MSCs with other cell types has also been applied as a means of influencing cellular behavior.^[^ ^40^, ^43^ ^]^ For example, the co‐culture of hypoxia conditioned hADMSC with human hepatocytes has been shown to reduce apoptosis and lead to increased extracellular collagen production, along with downregulation of pro‐apoptotic genes.^[^ ^43a^ ^]^ A study comparing the effect of hypoxic MSC secretome to hypoxia conditioned MSC co‐culture revealed that expression of HIF‐1*α* was increased in cardiomyocytes treated with secretome as compared to co‐culture.^[^ ^43b^ ^]^ Moreover, cellular proliferation marker *Ki‐67*, along with RhoA was upregulated in secretome treatment as compared to co‐culture.^[^ ^43b^ ^]^ Hence, depending on the cell type, introduction of secretome from hypoxic MSCs may lead to higher modulation of cellular behavior in the target cell as compared to normoxic MSC secretome or even co‐culture with hypoxia conditioned MSCs (Table 3).

**Table 3:** Summarizes the different studies that have reported the effect of hypoxia on MSC secretome

Reference	Material	Conditions and device used	Cell Source	Medium/Growth Factors Used	Reported Result
Lotfinia et al.^[^ ^38^ ^]^	‐	Hypoxic culture (1% O_2_) for 24, 48 and 72 h in modular incubator chamber (BillupsRothenberg)	Human bone marrow derived MSC (hbMSC) and embryonic stem cell (ESC) derived MSC	DMEM with 2 × 10^−3^ m L‐glutamine and 0.1% human serum albumin	Stronger immunomodulatory properties of secretome collected from hypoxic culture conditions. Secretome from ESC derived MSC had higher immunomodulatory property compared to bMSC
Chang et al.^[^ ^40a^ ^]^	‐	Hypoxic culture (0.5% O_2_) for 24 h in proOx‐C‐chamber system (Biospherix)	hbMSC	DMEM‐low glucose supplemented with 10% FBS	Hypoxia pre‐conditioned MSCs induced HGF and VEGF expression in medium
Zhao et al.^[^ ^40b^ ^]^	12‐well plate	Hypoxic culture (1% and 3% O_2_) for 7 days in two‐gas incubator (Thermo Scientific, Forma Steri‐Cycle i160 STERI‐cycle)	WJMSC (8 × 10^4^/well) co‐cultured with umbilical cord blood‐derived CD34+ cells (4 × 10^4^/well)	H5100 medium with 10^−6^ m hydrocortisone	Increased VEGF secretion and better maintenance of stemness of hematopoietic stem cells upon co‐culture
Teixeira et al.^[^ ^40c^ ^]^	T75 T‐flasks coated with gelatin for expansion; DASGIP Parallel Bioreactor system	Hypoxic culture (5% O_2_) for 7 days in DASGIP Parallel Bioreactor system	WJMSC; Cell density 24 000 cells mL^−1^	‐	Hypoxic pre‐conditioning upregulated several neuroregulatory proteins in secretome.
Xia et al.^[^ ^33^ ^]^	150 mm petri dish	Hypoxic culture (5% O_2_) for 48 h	ADMSC	DMEM‐low glucose supplemented with 0.8% FBS	Secretome from hypoxic ADMSCs promoted healing of the gastric mucosa in rats. Several proteins such as VEGF upregulated under hypoxia.
Qin et al.^[^ ^43a^ ^]^	Collagen‐coated microplates	Hypoxic culture (2% O_2_) for 24 h in hypoxia incubator chamber (StemCell Technologies)	hADMSC; Cell density: 20 000 cm^−2^ co‐culture with human hepatocytes	Low‐serum MSC expansion medium (Invitrogen)	Hypoxic culture improved extracellular collagen deposition along with downregulation of pro‐apoptotic genes.
Kastner et al.^[^ ^43b^ ^]^	Trans‐well plates	Hypoxic culture (<1% O_2_) for 2 h in hypoxia chamber (Billups‐Rothenberg Inc)	hbMSC co‐culture with human cardiomyocytes	DMEM high glucose with 10% FBS. Replaced with M199 medium 24 h prior to experiment	Increased expression of HIF‐1*α*, cellular proliferation marker *Ki‐67*, along with RhoA in cardiomyocytes treated with hypoxic MSC secretome compared to hypoxia conditioned MSC co‐culture.

## 3 Differentiation under Hypoxic Conditions

### 3.1 Adipogenic and Osteogenic Differentiation of MSCs Under Hypoxia Yields Varying Results

Studies exploring the differentiation of human and murine MSCs into adipogenic and osteogenic lineages under hypoxia have reported substantially varying results, with both stimulating and ameliorating results being reported (**Table** **4**, Figure 2). Significant impairment of osteogenic differentiation under hypoxia, observed by downregulation of osteogenic markers osteocalcin,^[^ ^27^, ^44^ ^]^ alkaline phosphatase activity (ALP) genes^[^ ^44^, ^45^ ^]^ and runt‐related transcription factor 2 (RUNX2),^[^ ^27^, ^46^ ^]^ reduced ALP activity^[^ ^12^, ^44^, ^47^ ^]^ and calcium deposition^[^ ^12^, ^45^, ^47^ ^]^ has been reported. ALP gene expression and level of ALP activity can serve as indicators for osteogenic differentiation and bone formation.^[^ ^48^ ^]^ Adipocyte formation has been reported to be ameliorated under hypoxia, with downregulation of adipogenic markers FABP4 and LPL.^[^ ^5^, ^12^, ^19^ ^]^ In contrast, a multitude of studies has also reported enhanced osteogenic^[^ ^5^, ^17^, ^23^, ^49^ ^]^ and adipogenic differentiation^[^ ^18^, ^23^, ^29^ ^]^ of MSCs under hypoxia. Equal differentiation potential of MSCs cultured under both normoxia and hypoxia has also been observed.^[^ ^19^, ^26^, ^29^, ^50^ ^]^

**Figure 3:**
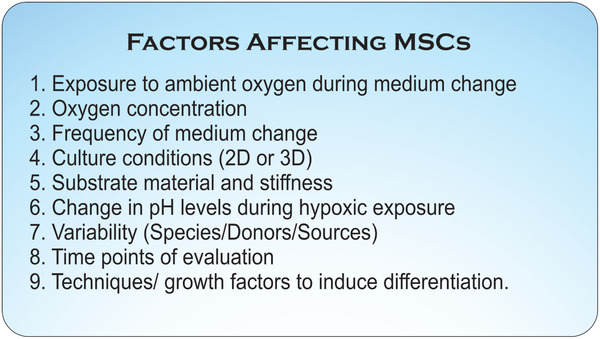
Factors affecting mesenchymal stem cell (MSC) behavior under hypoxia. These factors, both individually and in combination to yield synergistic effects, can affect MSC behavior and could explain the variable results reported in studies on the differentiation of MSCs under hypoxia. These factors should be considered during experimental design to avoid influence of these factors on the results.

These contrasting reports may be explained by variation in experimental design between studies, consisting of differences in species, exposure time to hypoxia and O_2_ concentration, substrates on which MSCs are cultured (alginate pellets, monolayers or scaffolds), techniques and growth factors used to induce differentiation and time points of evaluation (**Figure** **3**). Furthermore, it has been suggested that as the physiological O_2_ concentration on the bone surface varies in the range 5–12%, differentiation studies carried out at lower O_2_ concentrations could reduce osteoblastic differentiation.^[^ ^49^ ^]^

Preconditioning of MSCs under hypoxic conditions can lead to the upregulation of multipotency and could be a viable strategy to improve the differentiation potential of MSCs. Expansion of MSCs under hypoxia followed by differentiation under normoxia can increase the differentiation potential compared to differentiation under normoxia or hypoxia alone.^[^ ^12^, ^23^, ^45^, ^51^ ^]^ However, the duration of exposure to hypoxia and O_2_ concentration required to attain the highest differentiation potential is yet to be analyzed.^[^ ^24^ ^]^

**Table 4:** Summarizes the studies that apply hypoxia for osteogenic and adipogenic differentiation of mesenchymal stem cells

Reference	Material	Conditions and device used	Cell Source	Medium/Growth Factors Used	Reported Result
Burian et al.^[^ ^24^ ^]^	2D cell culture flask and 3D tricalcium phosphate (TCP) scaffolds with PHB	Hypoxic culture (2% O_2_) in humidified incubator (MCO‐5M, Sanyo) for 21 days	Porcine bone marrow‐derived MSC (pbMSC) and porcine adipose‐derived MSC (paMSC) Cell density 5 × 10^3^ cells mL^−1^	Osteogenic medium (DMEM with 10% FBS, 100 × 10^−9^ m dexamethasone, 50 × 10^−6^ m ascorbic acid 2‐phosphate and 10 × 10^−3^ m *β*‐glycerophosphate disodium)	Hypoxia attenuated osteogenic differentiation of pbMSCs compared to normoxia, and slightly increased osteogenic differentiation in paMSC
Volkmer et al.^[^ ^45^ ^]^	6‐well plate	Hypoxic culture (2% O_2_) for 21 days in multigas incubator;	hMSC Cell density 3000 cells cm^−2^	Osteogenic medium (High glucose DMEM supplemented with 10% FBS, 100 × 10^−9^ m dexamethasone, 10 × 10^−3^ m b‐glycerophosphate, 50 × 10^−3^ m l‐ascorbic acid 2‐phosphate and antibiotics).	Hypoxia increased proliferation of hMSCs but inhibited osteogenesis. Hypoxic preconditioning prior osteogenesis restored osteogenic potential.
Boyette et al.^[^ ^5^ ^]^	6‐well or 24‐well plates (osteogenic differentiation);	Hypoxic culture (5% O_2_) for 21 days in closed incubators;	hbMSC Cell density 1 × 10^4^ cells cm^−2^	Osteogenic medium (DMEM supplemented with 10% (FBS), 50 µg mL^−1^ L‐ascorbate‐2‐phosphate, 0.1 × 10^−6^ m dexamethasone, 10 × 10^−3^ m *β*‐glycerophosphate, and 10 × 10^−9^ m 1*α*,25‐(OH)_2_ vitamin D_3_)	Hypoxia during differentiation upregulated osteogenesis associated genes, alkaline phosphatase activity and total mineral deposition in hbMSCs.
Sheehy et al.^[^ ^49^ ^]^	6‐well plates (osteogenic differentiation)	Hypoxic culture (5% O_2_) for 14 days	pbMSC Cell density 3 × 10^3^ cells cm^−2^	Osteogenic Medium (DMEM GlutaMAX supplemented with 10% FBS, 20 µg mL^−1^ *β*‐glycerophosphate,100 × 10^−9^ m dexamethasone and l‐ascorbic acid‐2‐phosphate and antibiotics)	Osteogenic potential and calcium accumulation higher when pbMSCs were both expanded and differentiated under hypoxia compared to normoxia.
Zhang et al.^[^ ^44^ ^]^	6‑well plates	Hypoxic culture (2% O_2_) for 14 days in three‑gas modular hypoxic incubator (IG750, Jouan)	Rat bone marrow‐derived MSC (rbMSC) Cell density 2 × 10^4^ cells cm^−2^	Osteogenic medium (DMEM supplemented with 10% FBS, 0.1 × 10^−3^ m dexamethasone, 10 × 10^−3^ m *β*‐glycerophosphate and 50 × 10^−3^ m ascorbic acid)	Hypoxia reduces osteogenesis, ALP activity and mRNA expression of osteocalcin, ALP and collagen I in rbMSCs
Liu et al.^[^ ^50^ ^]^	Tissue culture plates	Preconditioning with hypoxia (5% O_2_) for 6 h	rbMSC Cell density 5 × 10^3^ cells cm^−2^	Osteogenic medium (DMEM supplemented with 10% FBS, dexamethasone, *β*‐glycerol phosphate and ascorbate); Adipogenic medium (DMEM supplemented with 1‐methyl‐3‐isobutylxanthine, dexamethasone, insulin, and indomethacin)	No difference in osteogenesis or adipogenesis of rbMSCs under hypoxia. Hypoxia enhanced survival of rbMSCs.
Lennon et al.^[^ ^17^ ^]^	Tissue culture plates	Hypoxic culture (5% O_2_) for 21 days in closed incubator chambers	rbMSC Cell density 5 × 10^7^ cells 100 mm^−1^	Osteogenic medium (Low‐glucose DMEM supplemented with 10% FBS,100 × 10^−9^ m dexamethasone, 80 × 10^−3^ m ascorbic acid 2‐phosphate, 10 × 10^−3^ m *β*‐glycerophosphate)	Hypoxia increased osteogenesis, with increased ALP activity and calcium content.
Basciano et al.^[^ ^23c^ ^]^	60 cm^2^ petri dishes	Hypoxic culture (5% O_2_) for 4 passages in incubator (Sanyo).	hbMSC Cell density 100 cells cm^−2^	Osteogenic medium (*α*‐MEM supplemented with 10% FBS, 2 × 10^−3^ m glutamine, 60 × 10^−6^ m ascorbic acid, 10 × 10^−3^ m *β*‐glycerol phosphate and 0.1 × 10^−6^ m dexamethasone)	Post expansion in hypoxia, cells showed higher potential for osteogenic differentiation with increased ALP and RUNX2 expression.
dos Santos et al.^[^ ^19b^ ^]^	12‐well plates	Hypoxic culture (2% O_2_) in C‐Chamber connected to Proox Model 21 controller (BioSpherix)	hbMSC Cell density 1000 cells cm^−2^	Osteogenic medium (Low glucose DMEM supplemented with 10% FBS, 100 × 10^−9^ m dexamethasone, 10 × 10^−3^ m *β*‐glycerophophate and 0.05 × 10^−3^ m 2‐phospho‐L‐ascorbic acid), Adipogenic medium (DMEM supplemented with 10% FBS, 170 × 10^−9^ m insulin, 0.5 × 10^−3^ m 3‐isobutyl‐1‐methyl‐xanthine, 0.2 × 10^−3^ m indomethacin, and 1 × 10^−3^ m dexamethasone)	No difference in osteogenic and adipogenic differentiation observed between cells expanded in hypoxia and normoxia.
Fehrer et al.^[^ ^12^ ^]^	6‐well plates	Hypoxic culture (3% O_2_) in Thermo Electron Corporation 3110 incubators	hbMSC Cell density 50 cells cm^−2^ for differentiation	Osteogenic medium (MEM supplemented with 20% FCS, 20 × 10^−3^ m *β*‐glycerol phosphate, 1 × 10^−9^ m dexamethasone, 0.5 × 10^−6^ m ascorbate‐2‐phosphate and antibiotics), Adipogenic medium (MEM supplemented with 20% FCS, 1 × 10^−6^ m dexamethasone, 50 × 10^−6^ m indomethacine, 0.5 × 10^−6^ m 3‐iso‐butyl‐1‐methylxanthine, 0.5 × 10^−6^ m hydrocortisone and antibiotics)	Reduced adipogenic differentiation and no osteogenic differentiation under both expansion and differentiation under hypoxia. Post expansion in hypoxia and differentiation in normoxia, cells showed higher osteogenic and adipogenic differentiation.
Malladi et al.^[^ ^47a^ ^]^	12‐well plates	Hypoxic culture (2% O_2_) for 15 days	Mouse adipose derived MSC (maMSC) Cell density 10 000 cells/well	Osteogenic medium (DMEM supplemented with 10% FBS, 100 µg mL^−1^ ascorbic acid, 10 × 10^−3^ m *β*‐glycerophosphate, antibiotics and with 1 × 10^−6^ m retinoic acid or 50 × 10^−9^ m vitamin D)	Hypoxia reduced osteogenesis, with decreased alkaline phosphatase activity and mineralization being observed.
Holzwarth et al.^[^ ^26^ ^]^	96‐well plates	Hypoxic culture (5%, 3% or 1% O_2_) for 14 days in Heracell gas addition incubators (Heraeus Instruments GmbH)	hbMSC Cell density 6250 cells cm^−2^	Basal medium (Low glucose DMEM supplemented with 5% human fresh frozen plasma, 10^7^ mL^−1^ platelets, 80 IU mL^−1^ heparin sulphate, 1 × 10^−3^ m glutamine and antibiotics), Adipogenic medium (Basal medium supplemented with 1 × 10^−6^ m dexamethasone, 60 × 10^−6^ m indomethacin, 0.5 × 10^−3^ m isobuthylmethylxanthine and 10 × 10^−6^ m insulin), Osteogenic medium (Basal medium supplemented with 10 × 10^−9^ m dexamethasone, 0.1 × 10^−3^ m L‐ascorbic acid‐2‐phosphate, 10 × 10^−3^ m *β*‐glycerol phosphate and 100 ng mL BMP‐2)	hbMSCs showed impaired adipogenic and osteogenic differentiation under 1% O_2_. Cells cultured at 3% O_2_ showed osteogenesis comparable to normoxia.
Fink et al.^[^ ^52^ ^]^	6‐well plates	Hypoxic culture (1% O_2_) for 3 days in In Vivo 400 hypoxic workstation (Maltec) for adipogenic differentiation	Immortalized hbMSC Cell density 2 × 10^5^ cells/well	Basal medium (EMEM with 10% FBS and antibiotics) for expansion; Adipogenic medium (DMEM supplemented with 10% FCS, 1 × 10^−6^ m dexamethasone, 0.45 × 10^−3^ m isobutyl methylxanthine, 170 × 10^−9^ m insulin, 0.2 × 10^−3^ m indomethacin, 1 × 10^−6^ m rosiglitazone and antibiotics)	hbMSCs showed morphological changes with cytoplasmic lipid inclusions under hypoxia, but adipocyte‐specific genes were not induced.
Ren et al.^[^ ^18^ ^]^	T25 culture flasks	Hypoxic culture (8% O_2_) for 7–8 days in modular airtight humidified chamber	mbMSC Cell density 1 × 10^5^ cells cm^−2^	Adipogenic medium (60% low glucose DMEM, 40% MCDB‐201 with 2% FBS and 2 × 10^–9^ m dexamethansone)	Cells showed 5‐ to 6‐fold increase in lipid droplets under hypoxia compared to normoxia. Hypoxia accelerated mbMSC proliferation and adipogenic differentiation.
Grayson et al.^[^ ^23d^ ^]^	Synthetic poly(ethylene terephthalate) (PET) fibrous matrices with 100 to 200 µm pore size	Hypoxic culture (2% O_2_) for 30 days in sealed chamber	hbMSCs Cell density 3 × 10^6^ cells per PET disk	Osteogenic medium (Basal medium supplemented with 100 × 10^−9^ m dexamethasone, 10 × 10^−3^ m sodium‐*β*‐glycerophosphate, and 0.05 × 10^−3^ m ascorbic acid‐2 phosphate), Adipogenic induction medium (High glucose DMEM with 10% FBS, 0.2 × 10^−3^ m indomethacin, 0.5 × 10^−3^ m isobutyl‐1‐methyl xanthine, 1 × 10^−3^ m dexamethasone, and 5 mg mL^−1^ insulin) for 2 days, Adipogenic maintenance medium (High glucose DMEM supplemented with 10% FBS and 10 mg mL^−1^ insulin)	Hypoxic hbMSCs expressed higher levels of osteogenic and adipogenic differentiation markers
Salim et al.^[^ ^23e^ ^]^	‐	Hypoxic (2% O_2_) or anoxic (<0.02% O_2_) culture for 24 h in hypoxia workstations (Bactron Anaerobic/Environmental Chamber)	hbMSC	Basal medium (Poietics MSCGM Mesenchymal Stem Cell Medium) for expansion, Osteogenic medium (Basal medium with 1 m dexamethasone, 5 × 10^−3^ m *β*‐glycerophosphate, and 100 g mL^−1^ ascorbic acid)	Post expansion under hypoxia, hypoxic hbMSCs showed osteogenesis comparable to normoxia. Anoxic culture inhibited osteogenesis, visualized by downregulation of Runx2 and extracellular calcium deposition.
Yang et al.^[^ ^46a^ ^]^	12‐well plates	Hypoxic culture (1% O_2_) for 3 days	hbMSC Cell density 1 × 10^4^ cells cm^−2^	Osteogenic medium (*α*‐MEM supplemented with 16.6% FBS, 50 mg mL^−1^ ascorbate‐2 phosphate, 10^–8^ m dexamethasone and 10 × 10^−3^ m *β*‐glycerophosphate)	Hypoxia inhibited osteogenesis of hbMSCs, visualized by downregulation of Runx2 and reduced staining by Alizarin Red as compared to normoxia.
Tamama et al.^[^ ^23f^ ^]^	24‐well plates for osteogenic and 6‐well plate for adipogeni differentiation	Hypoxic culture (1% O_2_) in hypoxia chamber (Stemcell Technologies)	Primary hMSCs Cell density 5 × 10^4^ cells/24‐well for osteogenic and 1 × 10^6^ cells/6‐well differentiation	Osteogenic medium (*α*‐MEM supplemented with 10% FBS, 100 × 10^−9^ m dexamethasone, 10 × 10^−3^ m sodium‐*β*‐glycerophosphate, and 0.05 × 10^−3^ m ascorbic acid‐2 phosphate), Adipogenic induction medium (High glucose DMEM with 10% FBS, 0.2 × 10^−3^ m indomethacin, 0.5 × 10^−3^ m isobutyl‐1‐methyl xanthine, 1 × 10^−3^ m dexamethasone, and 5 mg mL^−1^ insulin) for 2 days, Adipogenic maintenance medium (High glucose DMEM supplemented with 10% FBS and 10 mg mL^−1^ insulin)	HbMSCs showed decreased osteogenic and adipogenic differentiation under hypoxia. Hypoxia promoted hbMSC self‐renewal and maintained undifferentiated phenotype.
Huang et al.^[^ ^47b^ ^]^	6‐well plate	Hypoxic culture (2% O_2_) for 21 days in hypoxia incubator chambers (Thermo Fisher Scientific)	rbMSC Cell density 10 000 cells cm^−2^	Basal medium (High glucose DMEM with 10% FBS and antibiotics)	Hypoxia inhibited spontaneous calcification of rbMSCs alongwith decreased ALP expression and calcium content. Osteogenic differentiation markers were downregulated in hypoxia compared to normoxia.
Hu et al.^[^ ^29^ ^]^	35 mm petri dish	Hypoxic culture (5% O_2_ and 10% O_2_) for 14 days.	mbMSC	Osteogenic medium (DMEM with 10% FBS, 1% glutamine, 0.1 × 10^−6^ m dexamethasone, 10 × 10^−3^ m *β*‐glycerophosphate disodium salt hydrate, and 50 × 10^−6^ m L‐ascorbic acid 2‐phosphate sesquimagnesium salt hydrate), Adipogenic induction medium (DMEM with 10% FBS, 1% glutamine, 1 × 10^−6^ m dexamethasone, 0.125 × 10^−3^ m indomethacin, 0.5 × 10^−3^ m 3‐isobutyl‐1‐methyl‐xanthine, and 5 µg mL^−1^ insulin) for 3 days and adipogenic maintenance medium (DMEM with 10% FBS, 1% glutamine, and 1 × 10^−6^ m dexamethasone) for 1 day.	Hypoxia (5% O_2_) enhanced adipogenic differentiation, while mbMSCs in both hypoxia and normoxia showed similar osteogenic differentiation. No significant difference between normoxia and hypoxia when differentiation was carried out at 10% O_2_

### 3.2 Hypoxia Increases Chondrogenic Differentiation Potential of MSCs

Various studies have reported that exposure of both human and murine MSCs to hypoxic conditions increases the chondrogenic differentiation potential (**Table** **5**, Figure 2).^[^ ^5^, ^9^, ^30^, ^37^, ^46^, ^53^ ^]^ Enhanced chondrogenic differentiation was observed for pellet cultures^[^ ^5^, ^27^, ^28^, ^37^, ^47^, ^49^ ^]^ or in alginate beads^[^ ^25^, ^30^, ^46^ ^]^ at 2–5% O_2_ concentration, with upregulation of chondrogenic transcription factors L‐Sox5, Sox9 and Sox6. HIF‐1*α* regulates the chondrogenic transcription factor Sox by directly binding to it. During oxygen limitation, there is an upregulation of HIF‐1*α* within the cells, which leads to a corresponding upregulation of Sox.^[^ ^5^ ^]^

Substrate stiffness can play a vital role in influencing the chondrogenic differentiation potential of MSCs. When hMSCs were differentiated toward chondrogenic lineage on soft and stiff substrates, hypoxia induced higher upregulation of markers of chondrogenesis on soft substrates as compared to stiff substrates. Moreover, the cells showed spread morphology and formed colonies on the soft substrate.^[^ ^53^ ^]^

**Table 5:** Summarizes the studies that apply hypoxia for chondrogenic differentiation of mesenchymal stem cells

Reference	Material	Conditions and device used	Cell Source	Medium/Growth Factors Used	Reported Result
Duval et al.^[^ ^46b^ ^]^	Alginate beads	Hypoxic culture (5% O_2_) for 7 days in sealed chamber (Bioblock Scientific);	Human bone marrow‐derived mesenchymal stem cells (hbMSC) Cell density 5 × 10^6^ cells mL^−1^	alpha‐MEM supplemented with 10% fetal calf serum, 2 × 10^−3^ m L‐glutamine and antibiotics No exogenous growth factors added.	Hypoxia induced chondrogenesis in hbMSCs. Downregulation of osteogenic transcription factor Cbfa1/Runx2. Increased expression of chondrogenic transcription factors.
Foyt et al.^[^ ^53^ ^]^	Fibronectin coated polyacrylamide hydrogels	Hypoxic culture (2% O_2_) in incubator	hbMSC Cell density 3 × 10^4^ cells cm^−2^	Chondrogenic medium (High glucose DMEM supplemented with 2 × 10^−3^ m l‐Glutamine, 100 × 10^−9^ m dexamethasome, 1% ITS solution, 1% antibiotic, 50 µg mL^−1^ ascorbic acid‐2‐phosphate, 40 µg mL^−1^ l‐proline, and 10 ng mL^−1^ TGF‐*β*3)	Hypoxia upregulates markers of chondrogenesis in hbMSCs on soft substrates compared to stiff substrates (spread morphology, form colonies).
Boyette et al.^[^ ^5^ ^]^	Pellet culture	Hypoxic culture (5% O_2_) for 21 days in closed incubators;	hbMSC Cell density 1 × 10^4^ cells cm^−2^	Chondrogenic medium (Serum‐free DMEM supplemented with ITS Premix, 50 µg mL^−1^ ascorbic acid, 40 µg mL^−1^ l‐proline, 100 µg mL^−1^ sodium pyruvate, 0.1 × 10^−6^ m dexamethasone, and 10 ng mL^−1^ (TGF)‐*β*3)	Chondrogenesis was inhibited by preconditioning with hypoxia and when cells were both expanded and differentiated under hypoxia. In cultures expanded under normoxia, hypoxia applied during subsequent pellet culture enhanced chondrogenesis, observed by increase in pellet size and higher Alcian Blue and Safranin‐O/Fast Green staining.
Sheehy et al.^[^ ^49^ ^]^	Pellet culture and 2% agarose	Hypoxic culture (5% O_2_) for 14 days	pbMSC Cell density 3 × 10^3^ cells cm^−2^	Chondrogenic medium (High glucose DMEM GlutaMax supplemented with 100 µg mL^−1^ sodium pyruvate, 40 µg mL^−1^ l‐proline, 50 µg mL^−1^ l‐ascorbic acid‐2‐phosphate, 1.5 mg mL^−1^ BSA, 1 × ITS, 100 × 10^−9^ m dexamethasone, 2.5 µg mL^−1^ amphotericin B, 10 ng mL TGF‐*β*3 and antibiotics).	Enhanced chondrogenesis was observed when pbMSCs were differentiated under hypoxia in both pellets and hydrogels.
Malladi et al.^[^ ^47a^ ^]^	Micromass culture in culture dishes	Hypoxic culture (2% O_2_) for 15 days	Mouse adipose derived MSC (maMSC) Cell density 1 × 10^7^ cells mL^−1^	Chondrogenic medium (DMEM supplemented with 1% FBS, 1% penicillin‐streptomycin, 37.5 µg mL^−1^ ascorbate‐2‐phophate, ITS premix and 10 ng mL^−1^ TGF‐*β*1),	maMSCs showed decreased chondrogenesis under hypoxia, assessed by decreased production of collagen II and extracellular matrix proteoglycans.
Wang et al.^[^ ^25^ ^]^	Alginate beads	Hypoxic culture (5% O_2_) in a low oxygen incubator (NAPCO) for 14 days.	Human adipose derived MSC (haMSC) Cell density 4 × 10^6^ cells mL^−1^	Chondrogenic medium (Basal medium supplemented with 37.5 mg mL^−1^ ascorbate 2‐phosphate, 100 × 10^−9^ m dexamethasone, 5 mg mL^−1^ insulin, 5 mg mL^−1^ transferrin, 5 ng mL^−1^ selenious acid, 1 mg mL^−1^ bovine serum albumin, 4.28 mg mL^−1^ linoleic acid and 10 ng mL^−1^ TGF‐*β*1)	Hypoxia inhibited the proliferation of haMSCs but total collagen synthesis increased three fold, alongwith significant production of cartilage‐associated matrix molecules.
Krinner et al.^[^ ^28^ ^]^	Pellet culture	Hypoxic culture (5% O_2_) for 14 days in tri‐gas incubator (Thermo Fisher Scientific)	Sheep bone marrow derived MSC (sbMSC) Cell density 0.5 × 10^5^ cells/pellet	Chondrogenic medium (Chondrogenic Differentiation BulletKit supplemented with 10 ng mL^−1^ TGF*β*3)	sbMSCs showed increased chondrogenic differentiation and under hypoxia as compared to cells cultured in normoxia.
Adesida et al.^[^ ^37^ ^]^	Pellet culture	Hypoxic culture (3% O_2_) for 14 days	hbMSC Cell density 2.5 × 10^5^ cells/pellet	Chondrogenic medium (High glucose DMEM with 0.1 × 10^−3^ m nonessential amino acids, 1 × 10^−3^ m sodium pyruvate, 100 × 10^−3^ m HEPES buffer, 1 × 10^−3^ m sodium pyruvate, 0.29 mg mL^−1^ L‐glutamine, 0.1 × 10^−3^ m ascorbic acid 2‐phosphate, 10^–5^ m dexamethasone, 1x ITS+1 premix and 10 ng mL^−1^ TGF*β*1)	Exposure to hypoxia significantly increased chondrogenesis, observed by levels of glycosaminoglycans and by Safranin O staining. Upregulated expression of aggrecan, collagen II and Sox9 genes was also observed.
Henrionnet et al.^[^ ^30^ ^]^	Alginate beads	Hypoxic culture (5% O_2_) for 28 days	hbMSC Cell density 3 × 10^6^ cells mL^−1^	Chondrogenic medium (High glucose DMEM with 1% glutamine, 1% sodium pyruvate, 40 µg mL^−1^ proline, 10^–7^ m dexamethasone, 50 µg mL^−1^ ascorbic acid 2‐phosphate and 1 × 10^−3^ m CaCl_2_, 1% ITS and 10 ng mL^−1^ TGF‐*β*1)	Upregulation of chondrogenic markers SOX9, ACAN, COMP and COL2A1 when both expansion and differentiation carried out under hypoxia.

## 4 Effect of Hypoxic Culture Conditions on Cellular Behavior

### 4.1 Hypoxic Conditions can Lead to the Stabilization of HIF‐1*α*

Hypoxia leads to the stabilization and induction of HIF‐1*α* within the cells, further influencing Notch and Wnt/*β*‐catenin signaling and subsequently cell differentiation.^[^ ^26^, ^54^ ^]^ This protein has been found to strongly influence the metabolism, proliferation as well as the multipotency of MSCs.^[^ ^7^, ^22^ ^]^ For instance, HIF‐1*α* regulates the chondrogenic transcription factor Sox by directly binding to it.^[^ ^5^ ^]^ HIF‐1*α* has been found to rapidly degrade upon removal of hypoxic conditions, as the degradation of the protein is oxygen dependent, with a half‐life less than 1 min.^[^ ^55^ ^]^ This short life could affect the stability and expression levels of HIF‐1*α* within the MSCs during exposure to ambient environmental O_2_ concentration during medium change. MSCs showed increased proliferative and differentiative potential when medium change interval was 4 days compared to when medium was changed daily.^[^ ^29^ ^]^ The effect of exposure to ambient O_2_ could be reduced by degassing the culture medium prior to medium change and using individual wells, which could reduce the time cells are exposed to ambient O_2_.^[^ ^56^ ^]^

### 4.2 Extracellular pH may be Influenced by Hypoxic Conditions, Leading to Modulation of MSC Fate

Hypoxic cell culture conditions may lead to a decrease in the extracellular pH, termed as extracellular acidosis. Extracellular acidosis can lead to maintenance of stemness and attenuation of differentiation potential of MSCs.^[^ ^57^ ^]^ Increasing the pH level in conjunction with hypoxic treatment could mimic *in vivo* scenario during fractures, as fracture healing in vivo is associated with alkaline pH.^[^ ^24^ ^]^

### 4.3 Hypoxic Conditions Lead to Increased Oxidative Stress

Free radicals, namely reactive oxygen species (ROS) and reactive nitrogen species (RNS), are normal byproducts of cell metabolism and at low concentrations, these species are involved in beneficial functions such as immune function and cellular signaling.^[^ ^58^ ^]^ However, when a cell is exposed to a stress inducing stimulus, the cell produces free radicals at higher concentrations, which can have damaging effects.^[^ ^59^ ^]^ Hypoxic conditions lead to increased oxidative stress in cells due to increased levels of both ROS and RNS, and decreased expression of catalase enzyme, which is known to act as an antioxidant. Mitochondrial ROS production has been implicated in enhanced oxidative stress under hypoxia, with mitochondrial DNA‐depleted cells showing ablation of ROS production upon hypoxic treatment.^[^ ^60^ ^]^ The precise mechanisms by which hypoxic conditions lead to increase in oxidative stress via the mitochondria are not yet clear.^[^ ^61^ ^]^ While it is plausible that hypoxic conditions may lead to O_2_ generation in the mitochondria by slowing down electron transport, NO is also generated in the mitochondria during hypoxia, which might lead to increased concentrations of the oxidant ONOO^–^.^[^ ^61^ ^]^ O_2_ is the major free radical produced in the mitochondria under normoxia while under hypoxic conditions, ONOO^–^ is the major oxidant.^[^ ^61^ ^]^ Increase in ROS at physiological levels can be beneficial for hMSCs by inducing proliferation as these cells can effectively manage oxidative stress to a certain extent.^[^ ^62^ ^]^ Moreover, ROS have been implicated in regulation of MSC differentiation and controlling MSC cell fate.^[^ ^63^ ^]^ However, larger increases in ROS concentrations can result in a decrease in stem cell viability by arrest of cell cycle and subsequent apoptosis, especially when cells are exposed to acute hypoxia.^[^ ^7^, ^62^, ^64^ ^]^ Furthermore, ROS expression has been known to stabilize the expression of HIF‐1*α* in tumors, and it is plausible that a similar effect exists in MSCs.^[^ ^65^ ^]^

## 5 Effect of Culture Conditions on Results Observed after Exposure to Hypoxia

### 5.1 Variation in Experimental Setup and Culture Conditions Among Studies may Lead to Variation in Reported Results

Along with variation in O_2_ concentrations between different studies, a number of devices have been used for establishing hypoxic culture conditions in the various studies included in this review. These experimental setups may vary in terms of various characteristics, such as control over O_2_ concentration, with some devices offering precise and rapid modulation of the O_2_ concentration while other devices taking longer times to reach the desired concentration. Some devices can enable multi‐well plates to be exposed to hypoxic conditions, while others enable control over O_2_ concentrations in single wells. As mentioned in the previous section, a major challenge in experiments involving hypoxia is preventing O_2_ from the ambient environment from reaching the cells. O_2_ is a small molecule with fast diffusion rates and can quickly diffuse into the hypoxic setup. As some factors influenced during hypoxic culture conditions, such as HIF‐1*α* expression, have a half‐life less than 1 min, it is imperative to maintain a strongly regulated culture environment in terms of O_2_ concentration.^[^ ^66^ ^]^ While large incubators can enable exposure of large number of samples to hypoxia, convective forces during opening and closing of the incubator door, for example, for retrieval of samples for medium change or experiments can lead to a temporary variation in the O_2_ concentration and may lead to variable results.^[^ ^67^ ^]^ This temporal variation in O_2_ concentration could be reduced by employing devices that expose individual wells to hypoxia separately, at the cost of limited number of parallel samples. However, the O_2_ concentration experienced by cells is lower than the desired concentration owing to limited diffusion of O_2_ through the culture medium.^[^ ^66^ ^]^ In some cases, it may be possible that O_2_ from the ambient environment diffuses into the tubing supplying the hypoxic gas, which could be avoided by shielding the tubing from the ambient environment.^[^ ^66^ ^]^ Thus, in addition to sensitive tests to detect the effect of hypoxic culture conditions on MSCs, it is also important to maintain a strongly regulated culture environment to avoid variations in results (Figure 3).

### 5.2 Substrate Properties can Influence the Effect of Hypoxia on MSCs as Mechanosensitive Pathways may Modulate Factors Influenced by Hypoxia

Hypoxic cultures of MSCs have been carried out on various substrates such as alginate beads, in monolayers or in cell pellets, or in different culture conditions, such as 2D versus 3D culture (for example, culture in monolayers on TCP or as pellets encapsulated in alginate beads, or by seeding onto scaffolds). It has previously been shown that hypoxia can influence MSC differentiation on substrates with different stiffness. The effects brought about by hypoxia and substrate stiffness can have a combinatorial effect on MSCs as mechanosensitive pathways may modulate factors influenced by hypoxia. When hMSCs were differentiated toward chondrogenic lineage on soft and stiff substrates, hypoxia induced higher upregulation of markers of chondrogenesis on soft substrates as compared to stiff substrates. Moreover, the cells showed spread morphology and formed colonies on the soft substrate.^[^ ^53^ ^]^ The behavior of MSCs under hypoxia could be compared in both 2D and 3D cultures, such as by using hydrogels or scaffolds. 3D cultures, coupled with hypoxia, could lead to a more physiologically relevant cellular environment with characteristic chemical and biophysical interactions with their environment as well as with other cells in the surrounding space.

### 5.3 MSC Behavior Under Hypoxia can Vary Depending on the Cell Source

Furthermore, it has also been reported that the morphology of MSCs under hypoxia can be influenced by the donor from whom the cells were isolated, with varying effects observed between MSC cultures from different donors under the same culture conditions. Under similar hypoxic conditions, MSCs isolated from different paediatric donors showed different morphologies, along with slight variation in differentiation potential.^[^ ^26^ ^]^ In addition, the age and biological sex of MSCs can impact cell fate, including the proliferation and differentiation potential.^[^ ^68^ ^]^

In addition to donor‐dependent variation in MSC characteristics, MSCs isolated from different sources or tissues in the body can further have varying response to hypoxic culture conditions. hMSCs isolated from bone marrow, adipose tissue, umbilical cord blood and amniotic fluid proliferate at different rates under hypoxia. Cells from amniotic fluid and umbilical cord blood proliferated significantly faster under hypoxia compared to other sources.^[^ ^10^ ^]^ Such differences can also be observed in the cell secretome, with medium from hypoxic preconditioned MSCs derived from ESCs showing stronger immunomodulatory effects as compared to medium from MSCs derived from bone marrow.^[^ ^38^ ^]^ It is plausible that variation due to these factors might also be reflected in the variable outcomes reported in previous studies, and a comparative study involving MSCs of multiple age and from different sources could provide deeper insight into the magnitude of effect of these factors (Figure 3).

## 6 Combining Hypoxic Conditions with Other State of the Art Techniques

**Figure 4:**
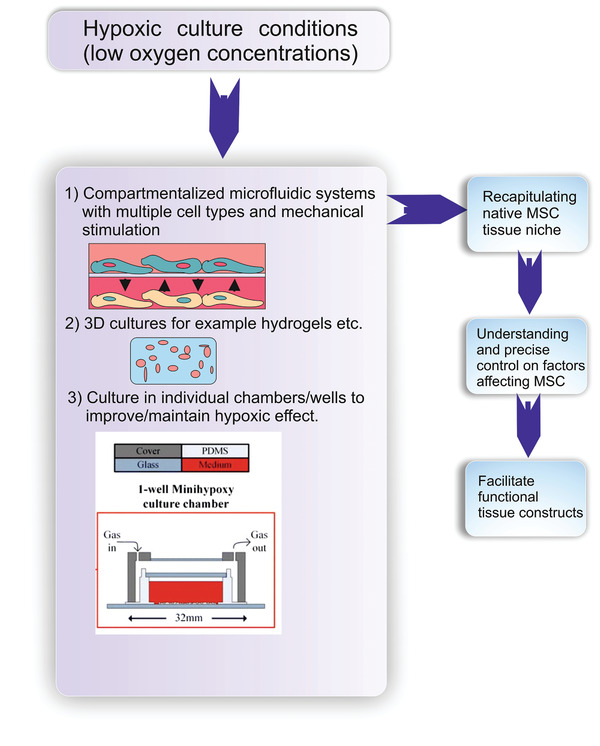
Future Perspective: Hypoxic conditions could be combined with other cell culture techniques such as microfluidic systems and 3D cultures to better recapitulate the mesenchymal stem cell (MSC) tissue niche and lead to better understanding and possibly precise control on factors affecting MSC fate. Image in Part 3) adapted under Creative Commons License from Metsälä et al.^[^ ^56^ ^]^

Microfluidic systems allow controlled exposure of cells to nutrients, growth factors and morphogens, thereby enabling precise spatiotemporal manipulation of the cellular microenvironment.^[^ ^69^ ^]^ Microfluidic systems coupled with hypoxia could allow for precise control of various factors experienced by MSCs, while circumventing problems such as exposure to ambient oxygen during medium change.^[^ ^70^ ^]^ Previous studies have employed flow of oxygen scavengers such as sodium sulfite in the channels, incorporated on‐off gas mixers onto microfluidic chips or carried out chemical or electrolytic reactions in channels to generate O_2_ gradients as a means to control the O_2_ concentration and generate hypoxic culture conditions coupled with microfluidics.^[^ ^71^ ^]^ Furthermore, compartmentalized microfluidic systems involving multiple cell types allowing for cell to cell interaction,^[^ ^72^ ^]^ along with hypoxic culture conditions and even mechanical stimulation, could enable the recapitulation of the MSC tissue niche ( **Figure** **4**).

*In vivo*, MSCs are found in a 3D environment, with distinct cell‐cell and cell‐matrix interactions that are lacking in conventional 2D monolayer cultures.^[^ ^73^ ^]^ Application of 3D cultures, such as hydrogels, to MSCs, in conjunction with hypoxic conditions, might enable the in vitro recapitulation of the native cellular microenvironment and could lead to the development of robust model systems for studying disease progression, drug screening, therapeutic applications and even for potential application in regenerative medicine. Variations between 2D and 3D cultures under hypoxia are evident in studies involving chondrogenesis, with MSCs in 3D cultures showing enhanced chondrogenic potential as compared to monolayer cultures.^[^ ^30^ ^]^

Maintaining an uninterrupted and resilient hypoxic environment is challenging, especially with longer cell culture durations.^[^ ^66^ ^]^ Culturing MSCs in individual chambers/wells could be an effective approach in circumventing the challenge. This could reduce the duration of exposure of MSCs to ambient O_2_ during various experimental steps such as medium change and imaging, and also form a robust system in which exposure of one well to ambient O_2_ may not affect cells in other wells. During medium change of a multi‐well plate, for example a 12‐well plate, it is plausible that the O_2_ concentration in the last well may increase due to longer exposure of cells in the last well to O_2_ in the ambient environment. As factors influenced during hypoxia, such as hypoxia inducible factor, have very short half‐life, this might even lead to variation in results between wells of the same well plate. Further studies are warranted in the future to observe the magnitude of effect this can have on cellular behavior.

## 7 Conclusion

Oxygen concentration plays an important modulatory role among nongenetic and environmental factors affecting MSC survival and plasticity in both in vitro and in vivo scenarios.^[^ ^74^ ^]^ Many studies have reported the ability of MSCs to proliferate and upregulate multipotency under prolonged hypoxic conditions (more than 24 h of hypoxic exposure), hinting that low O_2_ concentration may be an integral component of the native microenvironment experienced by MSCs. Studies on the differentiation of human and murine MSCs under hypoxia have been inconclusive, with widely varying results being reported, which could be due to the differences in species, exposure time to hypoxia and O_2_ concentration, physical constraints and cell‐cell/cell‐substrate interactions imparted by the culture conditions, such as 2D versus 3D culture (for example, culture in monolayers on TCP or as pellets encapsulated in alginate beads, or seeding onto scaffolds), techniques and growth factors used to induce differentiation and time points of evaluation.

In conclusion, several factors in cell culture such as the frequency of medium change, scaffold properties and stiffness, soluble factors, pH etc. influence the effect of hypoxia, in parts, on stem cells and therefore stringent control on culture condition is necessary to obtain consistent results. (Figure 3). Understanding these factors and culture conditions that regulate stem cell proliferation and stemness will assist in designing efficient strategies for in vitro cell expansion without affecting their expression and function.

## Conflict of Interest

The authors declare no conflict of interest.
